# Implementation of an Antibiotic Timeout at Veterans’ Affairs Medical Centers (VAMC): COVID-19 Facilitators and Barriers

**DOI:** 10.1017/ash.2021.7

**Published:** 2021-07-29

**Authors:** Jorie Butler, Joshua Judd, Cassie Goedken, Vanessa Stevens, Nui Brown, Michael Rubin, Matthew Goetz

## Abstract

Effective stewardship strategies such as an “antibiotic timeout” to encourage prescriber reflection on the use of broad-spectrum antibiotics are critical to reduce the threat of multidrug-resistant organisms. We sought to understand the facilitators and barriers of the implementation of the Antibiotic Self-Stewardship Timeout Program (SSTOP), which used a template note integrated into the electronic health record system to guide decision making regarding anti- methicillin-resistant *S. aureus* (MRSA) therapy after 3 days of hospitalization. We conducted interviews at 10 Veterans’ Affairs medical centers (VAMCs) during the preimplementation period (N = 16 antibiotic stewards) and postimplementation (N = 13 antibiotic stewards) ~12 months after program initiation. Preimplementation interviews focused on current stewardship programs, whereas postimplementation interviews addressed the implementation process and corresponding challenges. We also directly asked about the impact of COVID-19 on stewardship activities at each facility. Interviews were transcribed and analyzed using consensus-based inductive and deductive coding. Codes were iteratively combined into barrier and facilitator groupings. Barriers identified in the preimplementation interviews included challenges with staffing, the difficulties of changing prescribing culture, and academic affiliates (eg, rotating physician trainees). Facilitators included intellectual support (eg, providers who understand the concept of stewardship), facility support, individual strengths of antibiotic stewards (eg, diplomacy, strong relationships with surgeons), and resources such as VA policies mandating stewardship. By the postimplementation phase, all sites reported a high volume of COVID-19 cases. Additional demands were placed on infectious disease providers who comprise the antibiotic stewardship teams, which complicated the implementation of SSTOP. Many barriers and facilitators mentioned were similar to those identified during preimplementation interviews. Staffing problems and specific providers not “getting it [stewardship activities]” continued, whereas facilitators centered around strong institutional support. Specific pandemic-related barriers included slow down or stoppage of stewardship activities including curbing of regular MRSA screening practices, halting weekly stewardship rounds, and delaying stewardship committee planning. Pandemic-specific staffing problems occurred due to the need for “all hands on deck” and challenges with staff working from home, as well as being pulled in multiple directions, (eg, writing COVID-19 policies). Furthermore, an increase in antibiotic use was also reported at sites during COVID-19 surges. Our findings indicate that SSTOP implementation met with barriers at most times; however, pandemic-specific barriers were particularly powerful. Sites with strong staffing resources were better equipped to deal with these challenges. Understanding how the program evolves with subsequent COVID-19 surges will be important to support the broad implementation of SSTOP.

**Funding:** No

**Disclosures:** None

